# Supplementary material for: Notch3 Transactivates Glycogen Synthase Kinase-3-Beta and Inhibits Epithelial-to-Mesenchymal Transition in Breast Cancer Cells
Source: Cells. 2022 Sep 14;11(18):2872. doi: 10.3390/cells11182872 (PMC9497076; doi:10.3390/cells11182872)
Supplement: Supplementary file 1 [file cells-11-02872-s001.zip › cells-1765598-supplementary.pdf]

Table S1. Antibodies used in the study.

Antibody	Company/Clone Number	mono/poly	Dilution
Notch3	CST/D11B8	Rabbit/mono	1:1000
GSK3β	CST/27C10	Rabbit/mono	1:1000
β-catenin	CST/D10A8	Rabbit/mono	1:1000
E-cadherin	CST/24E10	Rabbit/mono	1:2000
Vimentin	CST/D21H3	Rabbit/mono	1:1000
p-β-catenin (Thr41/Ser37/Ser33)	CST/9561S	Rabbit/mono	1:1000
GAPDH	ZSGB-BIO/TA-08	Mouse/mono	1:3000
Notch3*#	abcam/ab23426	Rabbit /poly	1:400
GSK3β*#	abcam/ab93926	Mouse/mono	1:400
anti-rabbit IgG*#	CST/7074S		1:3000
anti-mouse IgG*#	CST/7076S		1:3000

*Used for immunofluorescence, #Used for immunohistochemistry

Table S2. The target sequences of small interfering RNA (siRNA) or short hairpin RNA (shRNA) used in the study.

Name	Target Sequences
Notch3 Sense	UAUAGGUGUUGACGCCAUCCACGCA
Notch3 Antisense	UGCGUGGAUGGCGUCAACACCUAUA
NC Sense	UUCUCCGAACGUGUCACGUTT
NC Antisense	ACGUGACACGUUCGGAGAATT
psi-U6.1/eGFP/shNC target sequence*	GCTTCGCGCCGTAGTCTTA
psi-U6.1/eGFP/shRNA-GSK3β target sequence*	GGATCATTTGGTGTGGTATAT

*shRNA plasmid

Table S3. PCR or Quantitative Real-Time PCR primers used in the study**.**

**Name**	**Sequences of Primer**
Notch3 Forward Primer	TGGCGACCTCACTTACGACT
Notch3 Reverse Primer	CACTGGCAGTTATAGGTGTTGAC
GSK3β Forward Primer	GGCAGCATGAAAGTTAGCAGA
GSK3β Reverse Primer	GGCGACCAGTTCTCCTGAATC
β-catenin Forward Primer	AAAGCGGCTGTTAGTCACTGG
β-catenin Reverse Primer	CGAGTCATTGCATACTGTCCAT
GAPDH Forward Primer	AAGGTCGGAGTCAACGGATTTG
GAPDH Reverse Primer	CCATGGGTGGAATCATATTGGAA
GSK3β 1-1 Forward Primer*	GCAGCAAGCCTCTGTATCAA
GSK3β 1-1 Reverse Primer*	GCAAGCTCTCAGACGCTAAA
GSK3β 1-2 Forward Primer*	GCCTTTCTGGAAGCTTTGTTC
GSK3β 1-2 Reverse Primer*	CCGAGTGTTGGCCTCTTC
GSK3β 2 Forward Primer*	AAGGCGAATTCACCCTTTCC
GSK3β 2 Reverse Primer*	CGATCAGCCTGAGAAACCAATTA
GSK3β 3 Forward Primer*	ACAAACGACGTCCGTGAT
GSK3β 3 Reverse Primer*	CCCACCCAGCCTTACAC
GSK3β 2 Subcloning wild Forward Primer#	CGAGCTCTTTCCGTTCGGCTACCTTC
GSK3β 2 Subcloning wild Reverse Primer#	TCCCCCGGGGGCCGATCAGCCTGAGAAA
GSK3β 2 Subcloning mutant Forward Primer#	TCTTTGGGCGTGGCTCAGCGTCACTTTCTA
GSK3β 2 Subcloning mutant Reverse Primer#	TAGAAAGTGACGCTGAGCCACGCCCAAAGA

*ChIP Primer #pGL3-GSK3β-enhancer Subcloning Primer

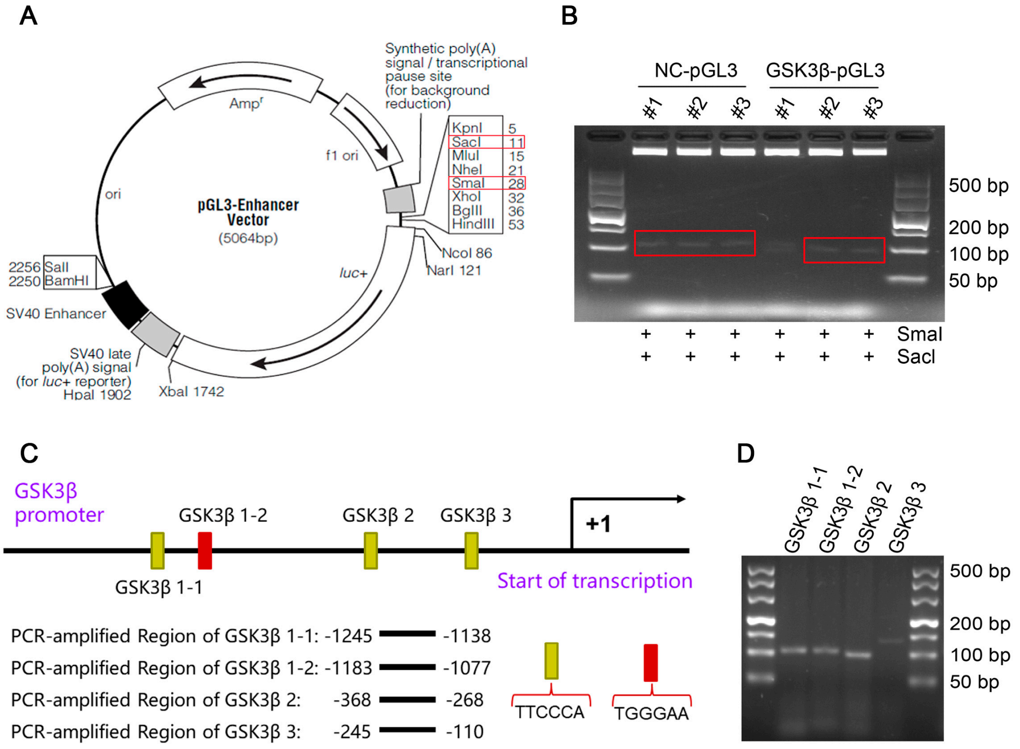

Figure S1. (A,B): The pGL3-GSK3β-Enhancer plasmid map. The plasmid architecture is pGL3-enhancer, and the inserted sequences are −354 bp to −268 bp upstream of the glycogen synthase kinase-3-beta (GSK3β) transcription start site, which is connected to SacI (5′-end) and SmaI (3′ -end), respectively (A). Three clones (1#, 2#, 3#) of both groups were digested with double enzymes; the target fragments were located correctly and the texture was identified successfully (B). (C): The GSK3β gene was analyzed and GSK3β promoter sequences were predicted using the UCSC Genome Browser website (http://genome.ucsc.edu/cgi-bin/hgGateway; last accessed on 28 October 2015); it was found that the promoter sequence contained a CSL-binding element regulating downstream of Notch3, and “TGGGAA” was located from −1131 bp to −1126 bp upstream of the transcription start site. Three antisense core sequences, “TTCCCA”, which were regulated by Notch3 downstream, were located at −1165 bp to −1160 bp, −308 bp to −303 bp, and −167 bp to −162 bp upstream of the transcription start site, respectively. Therefore, four primers were designed, among which GSK3β1-1 contained the first site, GSK3β 1–2 contained the first and second sites, GSK3β 2 contained site 3, and GSK3β 3 contained site 4. (D): Four primers were amplified by PCR and analyzed by agarose gel electrophoresis. All of these primers are available.

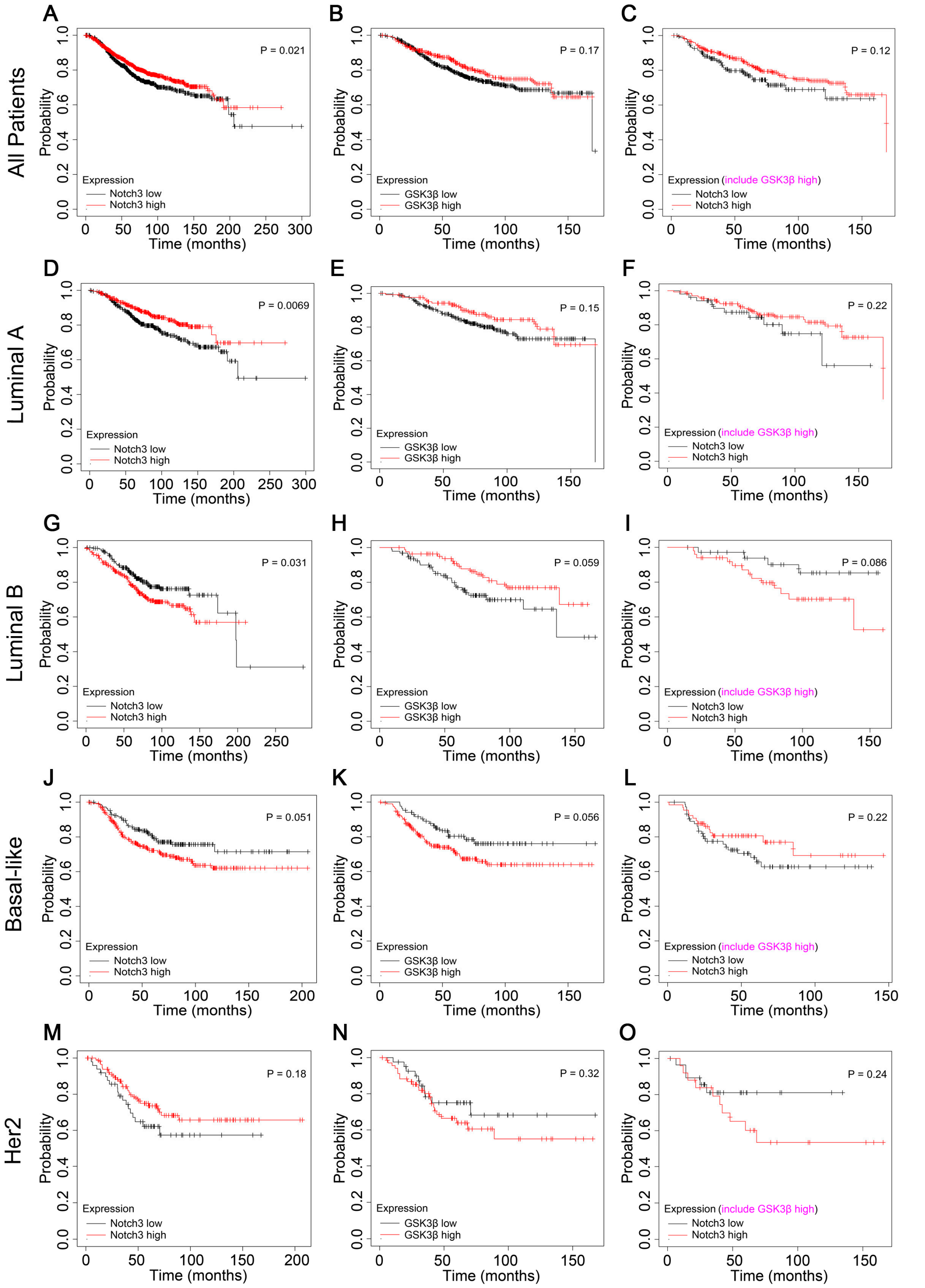

Figure S2. Relationship between OS, and Notch3 and GSK3β mRNA expression in breast cancer subtypes (A,D): The high expression of Notch3 was associated with better overall survival (OS) among different subtypes of breast cancer compared to the Notch3 low expression groups. (B,E,H,K,N): The OS of patients with breast cancer showing high expression of glycogen synthase kinase-3-beta (GSK3β) did not show a statistical difference compared to that of the GSK3β low expression groups. Notch3 levels predicted a worse OS in patients with luminal B (G) and basal-like (J) subtypes but did not affect the human epidermal growth factor receptor 2 (Her2) subtype (M). (C,F,I,L,O): Notch3 expression levels did not

affect the OS of all patients showing high GSK3β expression or those with high GSK3β expression in the different subtypes of breast cancer.
